# Hybrid graphene plasmonic waveguide modulators

**DOI:** 10.1038/ncomms9846

**Published:** 2015-11-10

**Authors:** D. Ansell, I. P. Radko, Z. Han, F. J. Rodriguez, S. I. Bozhevolnyi, A. N. Grigorenko

**Affiliations:** 1 School of Physics and Astronomy, University of Manchester, Manchester M13 9PL, UK; 2 Centre for Nano Optics, University of Southern Denmark, Campusvej 55, DK-5230 Odense M, Denmark

## Abstract

The unique optical and electronic properties of graphene make possible the fabrication of novel optoelectronic devices. One of the most exciting graphene characteristics is the tunability by gating which allows one to realize active optical devices. While several types of graphene-based photonic modulators have already been demonstrated, the potential of combining the versatility of graphene with subwavelength field confinement of plasmonic waveguides remains largely unexplored. Here we report fabrication and study of hybrid graphene–plasmonic waveguide modulators. We consider several types of modulators and identify the most promising one for telecom applications. The modulator working at the telecom range is demonstrated, showing a modulation depth of >0.03 dB μm^−1^ at low gating voltages for an active device area of just 10 μm^2^, characteristics which are already comparable to those of silicon-based waveguide modulators while retaining the benefit of further device miniaturization. Our proof-of-concept results pave the way towards on-chip realization of efficient graphene-based active plasmonic waveguide devices for optical communications.

## Abstract (web summary)

The combination of graphene with plasmonic waveguides remains largely unexplored. Here, Ansell *et al.* report the fabrication of hybrid graphene plasmonic waveguide modulators working in the telecom range, with a modulation depth greater than 0.03 dB μm^−1^ and with comparable characteristics to silicon-based devices.

## Graphical abstract

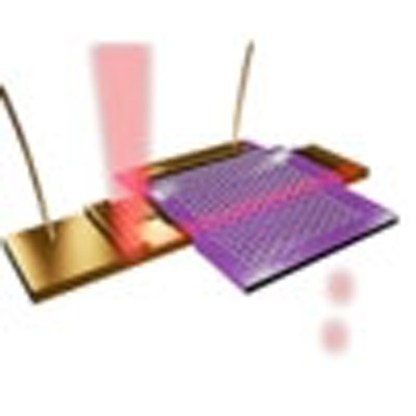

Graphene holds a great potential to provide efficient graphene-based photodetectors^1^,^2^,^3^,^4^, dedicated sensors^5^,^6^,^7^ and various optoelectronic devices^8^,^9^,^10^. Tunability of graphene conductivity by gating^11^,^12^,^13^,^14^ should allow one in principle to realize compact optical modulators. While several types of graphene-based photonic modulators have already been demonstrated^15^,^16^,^17^,^18^,^19^, the combination of plasmonic waveguides with graphene for the task of light modulation remains elusive. Hybrid graphene modulators^15^,^16^,^17^,^18^,^19^ promise unrivalled speed, low driving voltage, low power consumption and small physical footprints. Such modulators will be welcomed by telecommunications and optoelectronics. It is commonly assumed that the exceptional properties of graphene-based modulators are because of the fact that graphene is a material described by many superlative characteristics^20^: it is the thinnest, the strongest, with record thermal conductivity, showing the highest current density, completely impenetrable even for helium and with the highest charge mobility. Some of these extraordinary parameters, however, come about from dividing graphene characteristics by its extremely small thickness; a trick one performs to compare two-dimensional graphene with three-dimensional materials. In optics, for example, relatively small graphene absorption of ∼2.3% (observed at normal light incidence and defined by the fine structure constant^14^) could potentially result in extremely high modulation coefficient of 300 dB μm^−1^ (when exploiting optical Pauli blocking^9^) after normalization by graphene thickness, *t*_gr_=0.335 nm, is applied.

The small graphene thickness, while being great for obtaining superlative numbers, represents a significant hindrance for the tasks of active control of optical devices, since it implies that one graphene layer can affect only a small volume of propagating radiation, resulting in relatively small absorption and hence modulation induced by gating. In the transverse modulator configuration (that is, when radiation is incident on and transmitted through a graphene layer), the achievable modulation depth is just ∼0.1 dB per graphene layer. For waveguide modulators, in which a waveguide mode is propagating along and confined near a graphene plane (and which are the most promising for on-chip information processing), the portion of the waveguide mode interacting with the graphene layer is very limited^15^, depending strongly on the mode confinement. The resulting mode absorption coefficient (that represents the maximum modulation coefficient achievable via Pauli blocking) can be roughly estimated as follows: 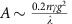 dB μm^−1^, where *n* is the waveguide effective refractive index, *γ* is the geometrical coverage factor, *λ* is the free space wavelength and *g*=*E*_in_/*E*_a_ represents the in-graphene-plane field component (responsible for the absorption by graphene) weighted with respect to the mode field (Supplementary Discussion 1). For typical parameters, *A* is at the level of 0.02–0.2 dB μm^−1^ in the general case of a hybrid graphene-waveguide modulator. Several graphene layers separated by dielectric can be used to improve this number^21^. However, it is difficult to arrange efficient gating for such a system and active optical devices normally involve a maximum of two graphene layers^17^.

To increase graphene interaction with light, one can use conventional metal plasmonics^3^,^9^,^22^,^23^,^24^,^25^,^26^. Plasmonic waveguides could provide smaller volumes of propagating modes (larger effective *n*) and local field enhancement (large values of *g*), which would result in higher modulation depth. There are two main problems in combining graphene and plasmonic waveguides to achieve optical modulation by gating: (i) the presence of metal layers complicates the task of gating with the spacer dielectrics being often affected by both high electric fields^27^ and light^28^ and (ii) it is difficult to realize plasmonic waveguide modes with large in-plane field components, since fields of surface plasmon polaritons (SPPs) supported by metal–dielectric interfaces are predominantly transverse in dielectrics^27^ (and the perpendicular electric fields do not excite currents in recumbent graphene). While the latter feature provides a useful possibility to safeguard metal plasmonics with graphene without degrading plasmon characteristics^24^, it makes the endeavour of designing hybrid graphene plasmonic waveguide modulators (HGPWMs) rather challenging—yet not hopeless. Simple estimations indicate the principal possibility of achieving strong SPP absorption by the graphene monolayer (even at telecom wavelengths), with the absorption coefficients reaching up to ∼2 dB μm^−1^ for strongly confined SPP modes (Supplementary Discussion 1B,C). At the same time, one should bear in mind that the SPP absorption by the graphene monolayer turns out to be quite similar in magnitude to that by adjacent metal because of Ohmic loss, as both absorption mechanisms are essentially determined by the same ratio between the transverse electrical field component in dielectric and the longitudinal one (in metal or graphene). To circumvent this very problematic issue (Supplementary Discussion 1), we suggest breaking natural coplanarity of graphene and metal layers so as to allow strong transverse SPP field components to have non-zero projections on a graphene layer.

Here we report the first HGPWM realization using different configurations, ranging from the simplest coplanar configuration that involves SPPs propagating along a metal–dielectric interface adjacent to a graphene monolayer, to the most efficient one based on wedge SPPs producing strong electrical fields along a graphene monolayer. To modulate the propagating SPP modes we have used the effect of optical Pauli blocking. This effect is based on the change of graphene optical conductivity induced by the shift of the Fermi energy produced by gating. To completely block optical transitions in graphene at the telecom wavelength of *λ*=1.5 μm, one needs to shift the Fermi energy from pristine values of *E*_F_≈0 to *E*_F_=*ℏπc*/*λ*≈0.4 eV.

## Results

### Hybrid graphene plasmonic waveguide geometry

**Figure 1:**
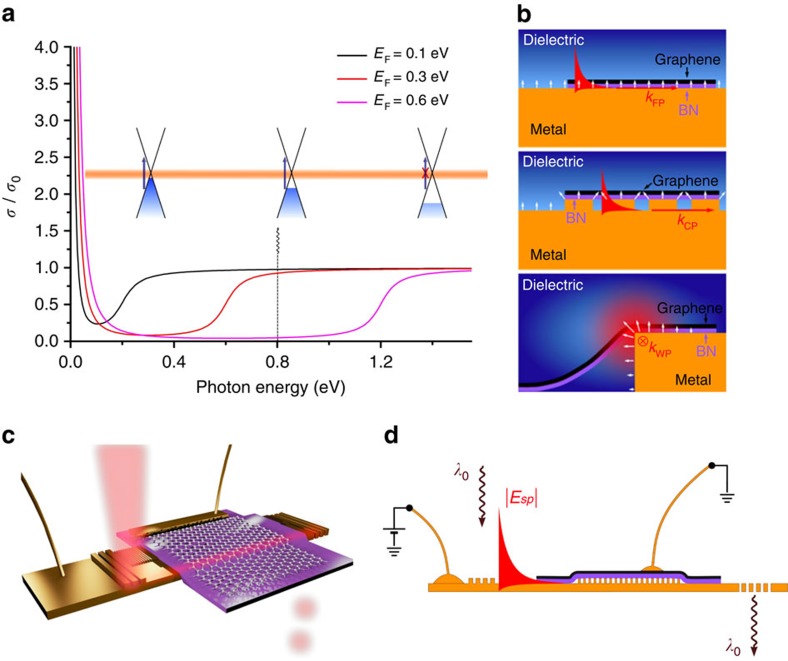
Principle of hybrid graphene plasmonic waveguide modulators. (**a**) Optical Pauli blocking expressed in terms of graphene relative conductivity. (**b**) Sketches of three types of plasmonic modes under investigation—flat, corrugated and wedge plasmons. White arrows indicate approximate direction of electric field. (**c**) 3D rendering of the experiment with the wedge plasmon mode. (**d**) The schematic of experiment where non-transparent grating couples light into plasmon modes (flat, corrugated or wedge), which can be affected by gated graphene, black layer, placed on the top of dielectric spacer (a flake of boron nitride, purple layer) and then be decoupled into light through the transparent grating.

The most straightforward HGPWM geometry modulated by the Pauli blocking effect (Fig. 1a) is based on the classical SPP configuration and is shown in Fig. 1b, top inset, where the gold strip (yellow colour), which supports the SPP propagation and serves as a backgate, is covered by a hexagonal boron-nitride (hBN) flake (purple colour), acting as a dielectric spacer and atomically smooth substrate for graphene, and a graphene flake (black colour). However, the SPP mode in such a waveguide configuration is, away from the surface plasmon resonance in the visible, only weakly bound to the metal surface and features primarily transverse electromagnetic fields, which do not excite currents in graphene, while in-plane fields (which do interact with the graphene in-plane conductivity) are negligibly small in the infrared^29^. Hence, the classical HGPWM configuration, producing only very weak graphene-related absorption (Supplementary Discussion 1A) and promising thereby only very weak modulation by gating, can hardly be used in practice. Attempting to enhance in-plane field components in the graphene layer, we introduced a nanostructured (corrugated) part of the plasmonic waveguide (Methods) so as to produce longitudinal near fields generated by the SPP mode propagating along the corrugated part of waveguide (see middle inset of Fig. 1b). It is however clear that the expected enhancement of in-plane field components is quite limited as the metal surface corrugation has to be shallow to not introduce significant additional propagation losses by scattering. Finally, we decided to make use of the wedge SPP mode supported by the edge of planar section of the waveguide^30^,^31^ (see bottom inset of Fig. 1b,c). This mode, in addition to enhanced in-graphene-plane fields near the edge of the strip that should result in higher modulation depth induced by graphene gating (Supplementary Discussions 1 and 5 and Supplementary Fig. 4), has superior field confinement characteristics, which is essential when considering potential applications to the surface plasmon circuitry. Figure 1d provides a general outline of modulation experiments: a non-transparent gold grating couples light into a plasmon-propagating mode that can be affected by gated graphene placed on the top of dielectric spacer and then running plasmons are decoupled into light through the transparent grating. Such configuration allows one to decrease the crosstalk between input and output lights, see Methods.

**Figure 2:**
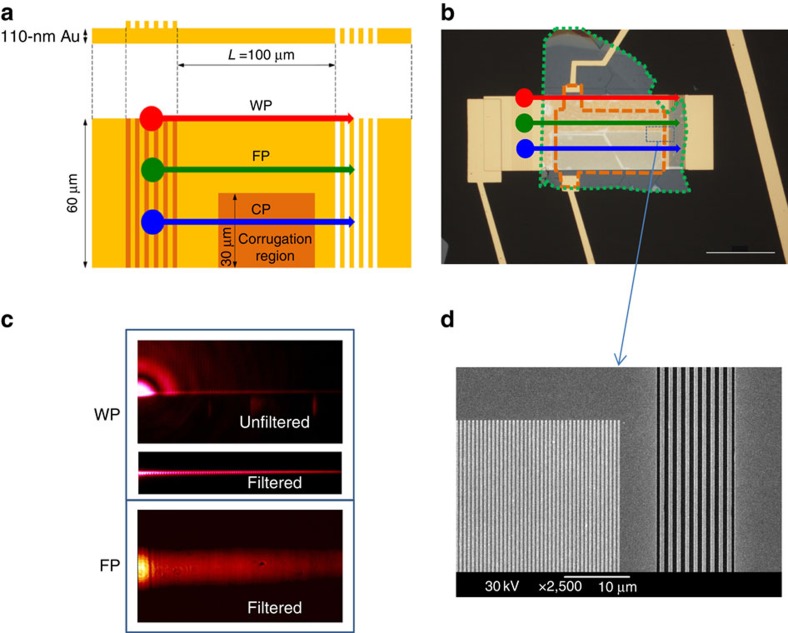
Plasmon modes of hybrid graphene plasmonic waveguide modulators. (**a**) The schematics of a studied plasmonic waveguide. The red, green and blue arrows represent WP mode, FP mode and CP mode, respectively. (**b**) The optical micrograph of a typical hybrid graphene plasmonic modulator studied in this work. The red, green and blue arrows represent WP, FP and CP modes, respectively. An area enclosed by green dotted line represents hBN. An area enclosed by dotted brown line represents graphene. Scale bar, 50 μm. (**c**) Leakage radiation detection of wedge, upper panel, and flat, lower panel, plasmon-propagating modes. The wedge mode is given in both raw and Fourier filtered images. (**d**) A scanning electron micrograph of an area shown in **b** by the dotted box that shows corrugated waveguide and the semitransparent decoupling grating.

All three studied plasmon–polariton modes—flat plasmons (FPs), corrugated plasmons (CPs) and wedge plasmons (WPs)—can be excited by moving the incident light beam to different parts of the coupler (Fig. 2a). An optical micrograph of one of our devices studied in this work is shown in Fig. 2b along with outlines demonstrating positions of hBN and graphene flakes. We have checked operation of plasmonic waveguides in both transmission and leakage radiation^32^,^33^ modes. Leakage radiation detection of plasmonic propagating modes for wedge and FPs are shown in Fig. 2c. Figure 2c confirms that the plasmonic modes were successfully excited and propagated along the waveguide. For completeness, Fig. 2d provides a scanning electron microscope micrograph of an area marked in Fig. 2b by the blue dashed box where the semitransparent decoupler and a part of the nanostructured area of the waveguide are shown. As preliminary experiments, we performed AFM studies of our samples to find thickness of hBN crystals (which turned out to be ∼50–70 nm, see Supplementary Fig. 1 and Supplementary Discussion 2) and hence to deduce the gating voltage necessary to induce optical Pauli blocking at the telecom wavelength (∼10–16 V). We also measured dc graphene resistance as a function of gating voltage in flat and corrugated regions of the waveguide with an idea to evaluate the position of the charge neutrality point (CNP) in graphene (*V*_CNP_∼0 V), see Supplementary Fig. 2 and Supplementary Discussion 3. Alternatively, the CNP has been evaluated from Raman measurements described in Supplementary Fig. 3 and Supplementary Discussion 4.

### Modulation of plasmonic waveguides by graphene gating

**Figure 3:**
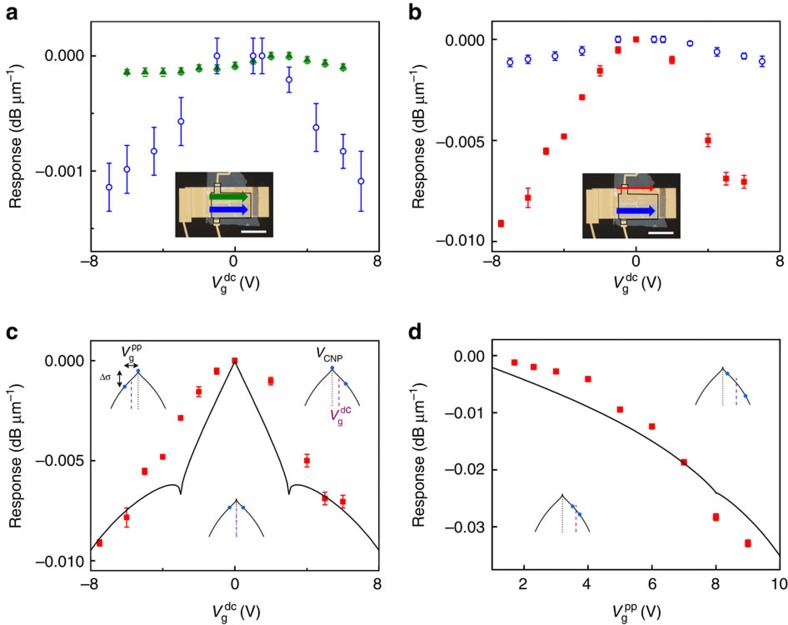
Operation of hybrid graphene plasmonic waveguide modulators. (**a**) Modulated AC transmission of the waveguide expressed in dB μm^−1^ as a function of gating voltage for the flat, green data points, and the corrugated, blue data points, plasmon modes. The peak-to-peak amplitude of AC modulation was 7.6 and 6 V for FP and CP modes, respectively, the frequency 6 Hz. The filled and empty data points represent two different devices. The inset shows the position where the plasmonic modes were measured. (**b**) Modulated AC transmission of the waveguide expressed in dB μm^−1^ as a function of gating voltage for the wedge, red data points, and the corrugated, blue data points, plasmon modes. The amplitude of AC modulation was 6 V, the frequency 6 Hz. Notice the 10-fold increase of the modulation signal for wedge mode. The filled and empty data points represent two different devices. The inset shows the position where the plasmonic modes were measured. (**c**) Theoretical fit to the experimental data for the wedge mode modulation as a function of gating obtained for the following parameters: peak-to-peak modulation amplitude 6 V, frequency 6 Hz. (**d**) Modulated AC transmission of the waveguide expressed in dB μm^−1^ as a function of AC peak-to-peak amplitude for the wedge plasmon mode, red data points, and theoretical fit to the experimental data. The dc offset was 6 V, the frequency 6 Hz. Error bars were estimated as a noise level in the absence of AC modulation and were combined with random errors whenever repeated measurements were performed.

Here we describe the main results of our experiments. The plasmonic waveguides were excited using telecom laser providing ∼3 mW of power at wavelength *λ*=1.5 μm. We measured the dynamic response of our modulators by applying an offset square-wave voltage to the back gate with peak-to-peak amplitude 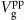 and dc component 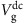. Figure 3 shows the modulation-depth characteristics both as a function of 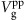 and 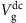. Comparing the modulation depth of FP and CP modes (Fig. 3a), one can see that the modulation effect is substantially stronger for CP: the CP mode gives around an eightfold increase in modulation depth compared with FP (for large 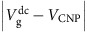). Here 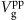 is set to 7.6 and 6 V for the measurements of the FP and CP mode modulation depths, respectively. For both FP and CP we see an approximately symmetrical increase in modulation depth with 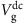, which we attribute to being the positions of the CNP (for the FP mode this is *V*_CNP_≈2.7 V; for the CP mode this is *V*_CNP_≈0.9 V). For other samples the modulation for FP modes compared with CP modes was even less pronounced (see Supplementary Fig. 5 and Supplementary Discussion 6). A drastic improvement in the modulation depth is expected from the increased interaction of graphene with electric field of the CP mode. Indeed, the longitudinal component of the electric field of FP is rather weak, whereas the presence of corrugations creates strong local longitudinal fields near ridges (see the sketch of the mode on Fig. 1b and, for example, ref. 34).

The WP mode provided us with the greatest modulation depths, achieved when probing the top edge of the waveguide, see inset Fig. 3b. Similarly to FP and CP, the modulation depth of WP increases symmetrically from an offset gate voltage 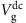, which in this case is ≈0 V and is close to *V*_CNP_ observed in dc resistivity measurements. For 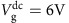 and 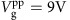 we were able to achieve a modulation depth of 3.3 × 10^−2^ dB μm^−1^, ∼30 and 230 times larger than the best CP and FP modulation depths with similar set parameters, respectively, Fig. 3d. It is important to note that the measured modulation depth for WP is underestimated, because, owingto the sample geometry, it was not possible to excite solely the WP mode—the FP mode was always excited on the adjacent flat region. As a result, the detected modulated WP signal had a contribution from FP, which was modulated by a substantially lower degree. A more than an order-of-magnitude increase in the modulation values of WP over CP (Fig. 3b,d) can be again attributed to an increased in-plane (in this case, lateral) electric-field component of the WP mode, which is essential in excitation of currents in graphene (see the sketch of the mode on Fig. 1b and Supplementary Fig. 4 and Supplementary Discussion 5 for a more detailed analysis of the WP mode). The largest modulation depth observed for the WP mode was ∼8.7% for a 12-μm modulation length. Note (see Supplementary Discussion 1) that the observed improvement in the modulation depth when changing from FP to WP configuration is quite similar to that estimated for the case of changing from the classical SPP configuration (∼0.002 dB μm^−1^) to the metal nanowire covered with the flat graphene layer (∼0.3 dB μm^−1^), that is, when exploiting the effect of metal surface curvature resulting in non-zero projections of strong transverse SPP fields on the graphene plane. The largest modulation depth of 0.03 dB μm^−1^ achieved in our hybrid graphene–plasmonic waveguides is lower than that (0.1 dB μm^−1^) reported for the hybrid graphene–silicon waveguides^15^. It is worth noting, however, that the silicon waveguide technology is significantly more matured than the plasmonic one. At the same time, our hybrid plasmonic waveguides are already made smaller than analogous silicon ones, while the modulation depth is expected to be considerably improved with a dedicated design.

## Discussion

We found that the dependency on 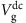 can be described using a simple theoretical model. Since our plasmonic waveguides are uniform along the propagation direction in the region of graphene flake, we can introduce an additional absorption coefficient induced by graphene as 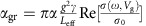, where *α* is the fine structure constant, *L*_eff_ is an effective size of plasmon mode, *σ*(*ω*, *V*_g_) is the gate-dependent graphene optical conductivity and *σ*_0_ is the universal graphene optical conductivity^14^. The additional dissipation of power introduced by the presence of graphene layer can be written as *P*=*P*_0_exp(−*α*_gr_*l*), where *l* is the length of the waveguide. Using the Kubo expression for graphene conductivity and writing Fermi energy as 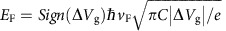, where Δ*V*_g_=*V*_g_−*V*_CNP_, *v*_F_ is the Fermi speed in graphene and *C* is the gate capacitance, we were able to model the modulation data obtained in our experiments. The modelling results (the solid lines of Fig. 3c,d) match our experimental data well. Insets in Fig. 3c,d provide a simplified picture of the change of modulation with gating. The square-wave gate voltage results in a switching between two optical conductivities, with the difference Δ*σ* dependent on 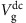 and 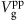 (see insets for Fig. 3c,d). When 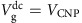, there is a switching between electron and hole conductivity, yet the magnitude of the absolute conductivity of graphene (and therefore transmission through the waveguide) is constant, and so the modulation depth is zero. As we move far enough from *V*_CNP_, Δ*σ* generally increases with 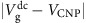 that leads to the observed dependency on 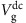. The scale of the modulation depth about *V*_CNP_ is then set by the magnitude of the coupling of graphene with the in-plane electric field of the plasmonic mode. Similar arguments explain the increasing modulation depth with 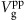 (Fig. 3d), when 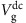 is set away from the CNP.

In conclusion, exploiting the enhancement of in-graphene-plane plasmonic fields provided by the wedge SPP waveguide configuration, we have demonstrated a prospective hybrid graphene–plasmonic waveguide modulator working at telecom wavelengths with reasonably high modulation depth of >0.03 dB μm^−1^ induced by a moderate gating voltage of <10 V. The modulator gating voltage can be reduced to several volts for hBN thickness of 10–20 nm. To achieve low gating voltages it is also important to have homogenous graphene flakes (that have constant doping along the flake surface). Note that the achieved modulation depth exceeds by ∼50% the intensity modulation depth corresponding to the highest phase modulation demonstrated with the state-of-the-art plasmonic modulator^35^. The frequency of the modulation is limited by the time constant of the structure^1^,^15^ and can be evaluated at the level >1 GHz. Plasmonic hybrid waveguides with a smaller physical size can provide even larger modulation speeds of >65 GHz (ref. 35). Therefore, one might expect that a waveguide based on cylindrical SPP (Supplementary Discussion 1C) could be modulated by graphene gating at frequencies of ∼100 GHz.

The use of wedge SPP waveguide configuration for light modulation by graphene gating thereby paves the way towards practical realization of very compact and efficient, potentially ultrafast^1^ and broadband^11^ hybrid graphene–plasmonic optical devices for a wide range of applications in optoelectronics and telecommunications.

## Methods

### Plasmonic waveguides

The gold plasmonic waveguides were fabricated in two lithography steps. In the first step of electron-beam lithography (and subsequent lift-off), we fabricated a plasmonic waveguide—a golden strip of 110 nm thickness, 100 μm length and 60 μm width—and the de-coupler, which was formed of a transparent gold grating for the period of 1.24 μm on the top of a quartz substrate. In the second step, we fabricated periodic lateral ridges along the bottom half of the waveguide (covering half of the width and the whole of the length of the gold strip) and the coupling non-transparent gold grating. The coupling grating was optimized for 1.5 μm wavelength normal incident light. This configuration of non-transparent coupling grating and transparent de-coupling grating allows one to excite the plasmon from one side and to measure the transmitted light from the other side, decreasing the crosstalk between input and output lights. Plasmons propagating along the nanostructured half of the waveguide, referred to as CP, hop over the ridges (arranged in a 600-nm-period grating) and generate in-plane electric fields, whereas along the planar section the evanescent electric field associated with the propagating FPs remains mostly transverse (Fig. 1b). We also studied the WP mode, which exists at the edge of planar section of the waveguide. Coupling of light and launching of plasmons was possible across the whole lateral dimension of the coupler, which allowed us to select the path of the propagating plasmons, see Fig. 2, and excite three different waveguide plasmon modes described above. Error bars in the experimental data were estimated as a noise level in the absence of AC modulation and were combined with random errors whenever repeated measurements were performed.

### Graphene preparation

To fabricate the modulation unit on the top of plasmonic waveguides, we applied the micromechanical cleavage technique to produce graphene and hBN flakes on oxidized silicon wafers. Then, hBN crystals and graphene were wet-transferred and aligned on to the waveguide. Oxygen plasma was used to shape graphene to achieve regular geometry of flakes across the waveguide. Here hBN acts as an atomically flat substrate for graphene as well as the gate dielectric. The gold waveguide was used both as the carrier of propagating plasmons and backgate for graphene gating. Finally, we fabricated electrical contacts to graphene (two terminals to allow for electrical characterization of our devices) and to the waveguide/backgate using photolithography. In this study we provide results obtained on two particular devices, with hBN thicknesses *t*≈50 and 70 nm and nanostructure ridge widths of *w*=300 and 200 nm, respectively. Both had a nanostructure ridge pitch of 600 nm.

## Additional information

**How to cite this article:** Ansell, D. *et al.* Hybrid graphene plasmonic waveguide modulators. *Nat. Commun.* 6:8846 doi: 10.1038/ncomms9846 (2015).

## Supplementary Material

Supplementary Information Supplementary Figures 1-5, Supplementary Discussion and Supplementary References.
