# Supplementary material for: Hybrid graphene plasmonic waveguide modulators
Source: Nat Commun. 2015 Nov 10;6:8846. doi: 10.1038/ncomms9846 (PMC5227092; doi:10.1038/ncomms9846)
Supplement: Supplementary Information — Supplementary Figures 1-5, Supplementary Discussion and Supplementary References. [file ncomms9846-s1.pdf]

Supplementary Information

Hybrid graphene plasmonic waveguide modulators

Supplementary Figures

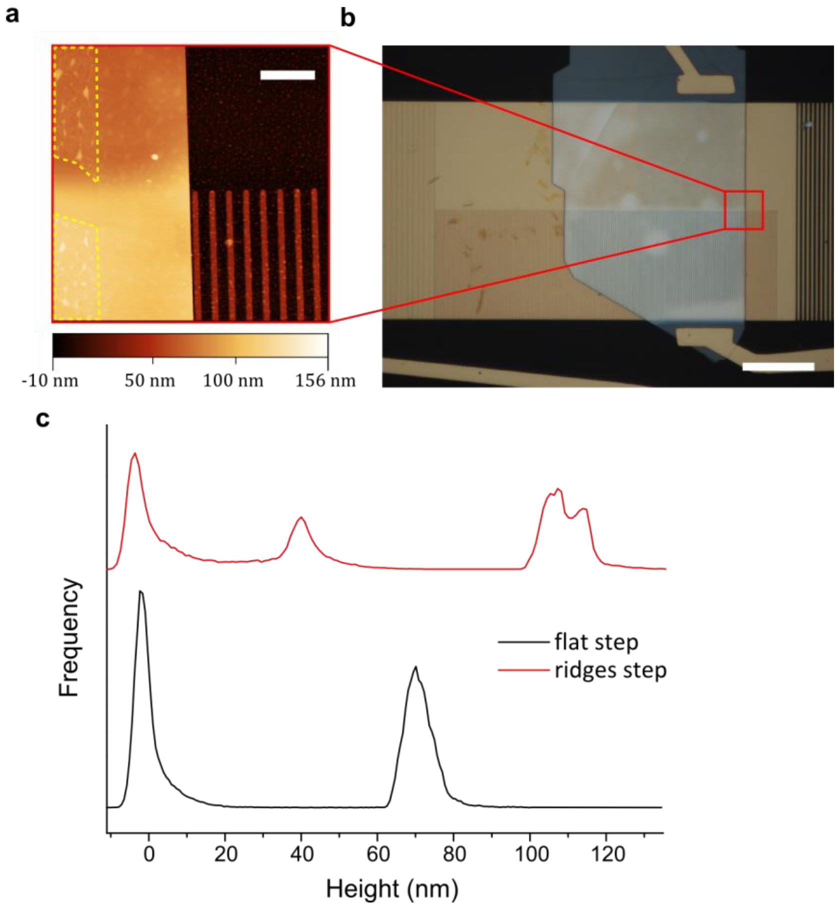

Supplementary Figure 1 | AFM topography measurement of device. a, Atomic force micrograph (scale bar: 2 μm) of region located in **b**. **b,** optical micrograph (scale bar: 20 μm) of one of our devices. No periodicity was detected for the ridges/hBN/graphene area, suggesting the heterostructure lies flat here. Graphene on hBN (dashed-yellow region) has a mesh-like appearance due to the aggregation of contaminants at the interface. **c,** Height distribution of measurements made in **a**: the 'ridges step' distribution samples the bottom half of the map; whilst the 'flat step' samples the top half of the micrograph.

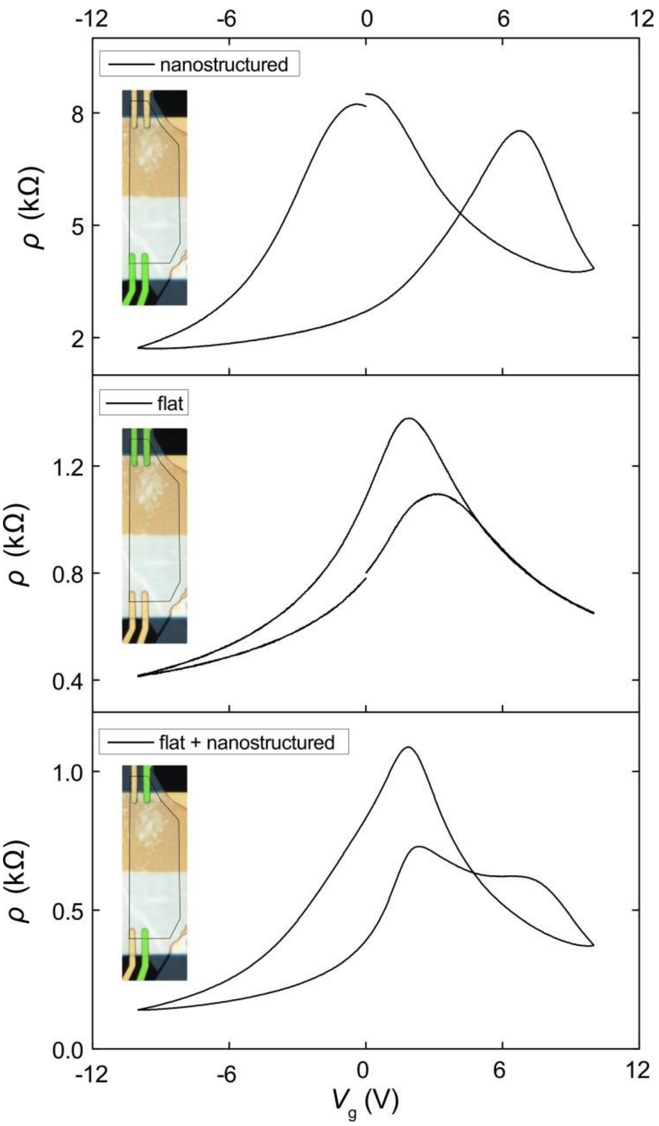

Supplementary Figure 2 | Resistivity measurements. Resistivity ρ of graphene as a function of gate voltage V_g measured on one of our devices. The terminal configuration allowed for measurements of charge carrier transport **a,** solely in the nanostructured region; **b,** solely in the flat region; and **c,** across the whole width of the device. Insets: optical micrograph of device, with thin outline of graphene added in black. Electrical contacts used for DC resistivity measurements are shown in false green.

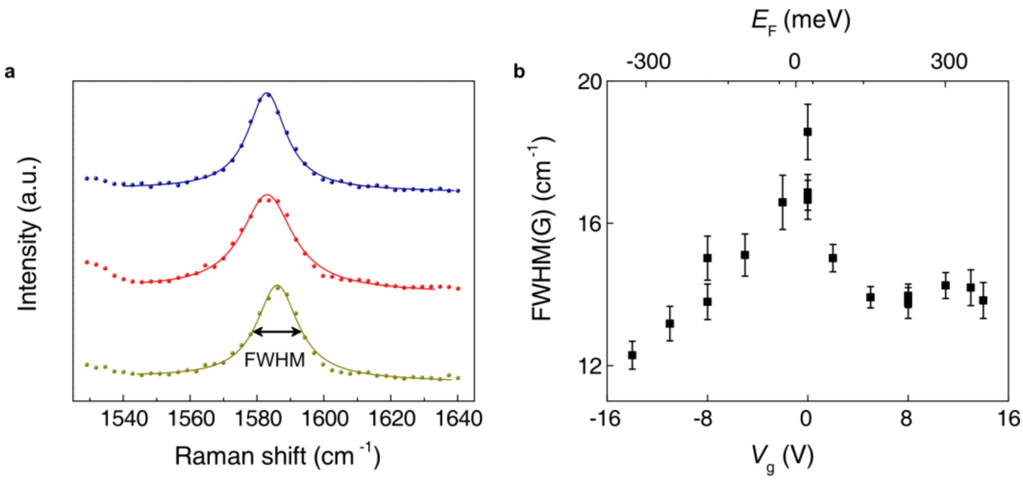

Supplementary Figure 3 | Raman spectroscopy of graphene. Measurements of FWHM of the G peak of graphene for different gate voltages on an analogous device. **a,** Raman spectra for $V_g = -5$ V (dark yellow), 0 V (red) and 5 V (blue). The symbols are the experimental data whilst the the solid lines are the fitted Lorentzian curves. **b,** FWHM of the G peak plotted as a function of V_g. The broadening of the FWHM of the G peak has a maximum at the CNP. The Fermi-energy scale has been set so this maximum is at $E_F = 0$ meV ($V_g = -0.95$ V).

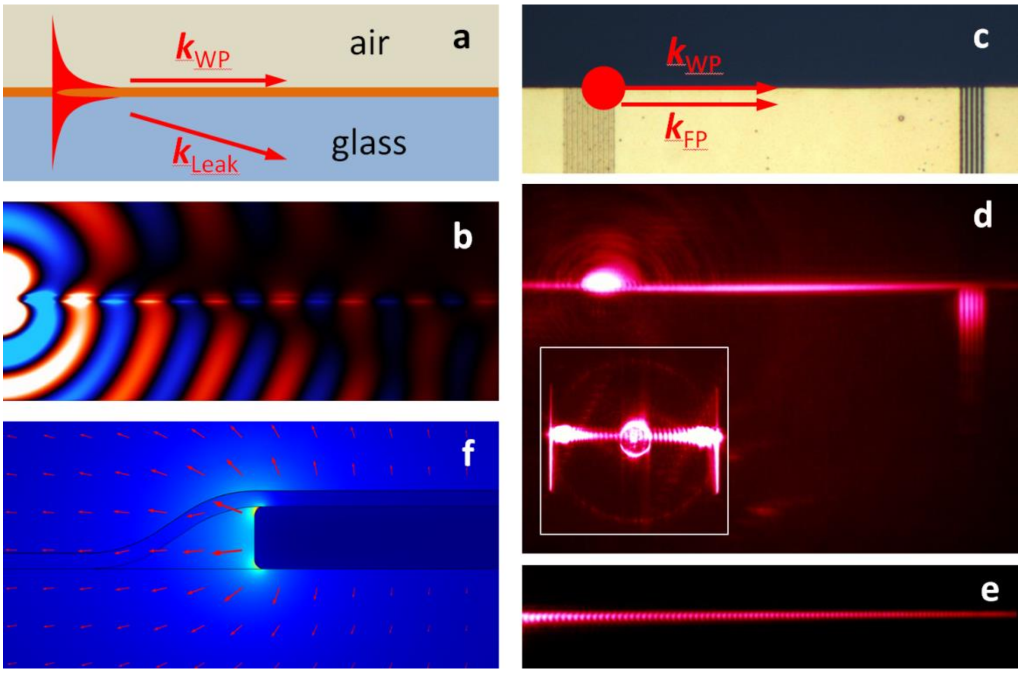

Supplementary Figure 4 | Wedge plasmon polariton mode. a, Schematics of WP mode propagating along an edge of a gold film (thickness 100 nm) separating glass and air media. **b,** Finite-difference time-domain modelling of the configuration shown on panel **a**. The plot shows *z* component of electric field. Excitation of WP is accomplished by *z*-polarized normally incident focused Gaussian beam. Leakage to the bottom (glass) substrate is immediately noticeable. **c,** Optical micrograph of a structure used for observation of WP mode. **d,** Raw and **e,** Fourier-filtered experimental images of WP mode taken in a leakage-radiation microscope. The inset on panel **d** is the corresponding Fourier-plane image. Vertical lines are the signature of confined waveguiding modes propagating in both (left and right) directions away from the in-coupling grating. **f,** Finite-element method modelling of the WP mode in the presence of BN and graphene layers wrapping the edge of the gold film. Red arrows show magnitude (proportional to their length) and direction of electric field of the mode. To simplify modelling, the refractive index of the substrate is taken to be equal to unity.

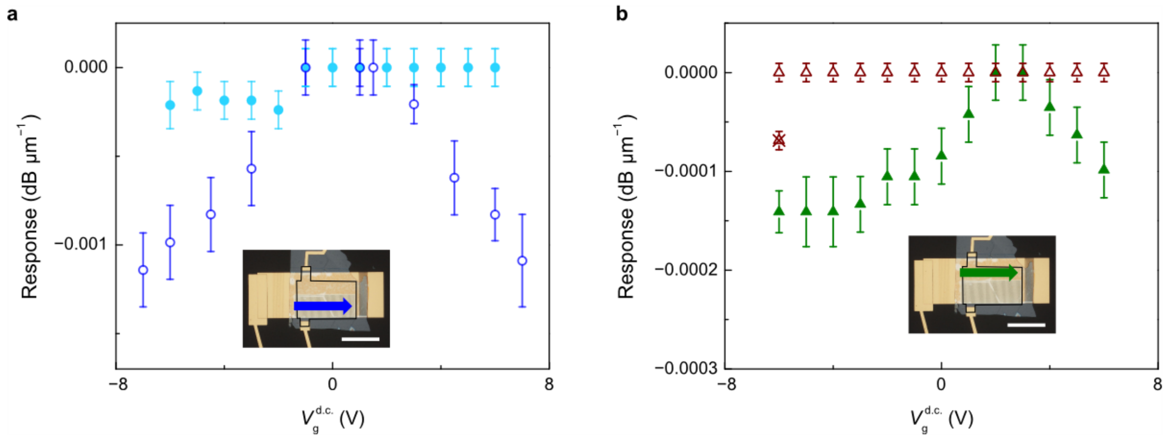

Supplementary Figure 5 | Operation of plasmonic modulators. a, Modulated transmission of the CP mode as a function of V_g^{dc} for two different devices, blue open circles (as described in the main manuscript) and light blue filled circles. The inset shows the position where the plasmonic modes were measured (all our devices had the same configuration. **b**, Modulated transmission of the FP mode as a function of V_g^{dc} for two different devices, light brown open triangles and green filled triangles. The crossed light brown triangle data point was taken on a separate day to the other light brown triangle data points.

Supplementary discussion

1. Graphene-based control of waveguide transmission

Let us consider a generic waveguide operating at the frequency ω and wavelength λ in free space. The distribution of electromagnetic field propagating along a waveguide, i.e., that of a waveguide mode, depends on a waveguide type and could be quite complex. In addition, plasmonic waveguides are subject to strong losses and strong dispersion. As a result, an introduction of an effective mode area for a waveguide (and other effective parameters!) is not simple and straightforward procedure[1-3]. For this reason, we will use crude approximations in order to evaluate the modulation depth one might hope to achieve in a waveguide using graphene gating (i.e., influencing the graphene absorption by Pauli blocking). We assume that the mode is propagating in the z-direction and is described by $\exp(i\beta z - i\omega t)$, where β is a complex constant. We also assume that one can introduce an effective mode area S, e.g., as[1]

$$S = \frac{1}{\mathrm{Max}\{W(\mathbf{r})\}} \int W(\mathbf{r}) dS \ , \tag{1}$$

where $W(\mathbf{r})$ is the energy density $\mathbf{r} = (x, y)$, and the effective mode width as[3]

$$L_{\mathrm{eff}}(y) = \frac{1}{\mathrm{Max}\{W(\mathbf{r})\}} \int W(\mathbf{r}) dx \,. \tag{2}$$

It is clear that, in general, only a part of a waveguide mode can be affected by graphene. For example, for a flat metallic waveguide, only a top surface of the metal can be physically covered with a graphene flake. For this reason we introduce a parameter γ which represents

graphene coverage of the mode perimeter. The parameter γ can be roughly defined as $\gamma = \frac{L_{\text{flake}}}{2\pi L_{\text{eff}}}$, where L_{flake} is the width of the flake perpendicular to the mode propagation direction and $L_{\text{eff}} = \text{Min}\{L_{\text{eff}}(y)\}$. The coverage parameter γ represents how accessible is the waveguide mode for graphene placement and how continuous is a graphene flake. For a circular plasmonic waveguide (metal nanowire) completely covered with a graphene flake γ ≅1, for a flat metal stripe completely covered with a graphene flake γ ≅0.5, etc.

Additional absorption of energy ΔW on the length Δz per time Δt induced by a graphene flake can be evaluated as

$$\Delta W \approx \text{Re}[\sigma] E_{\text{in}}^{2} \gamma 2\pi L_{\text{eff}} \Delta z \Delta t, \tag{3}$$

where Re[σ] is the real part of graphene conductance, E_{in} is the average local in-plane field along the graphene. The amount of energy which flows through the area at a position z per time Δt can be estimated as

$$W = C \cdot S E_{\text{a}}^{2} V_{\text{gr}} \Delta t, \tag{4}$$

where C is a constant, E_{a} is an average mode field and V_{gr} is the group velocity of the mode. This means that the ratio of graphene absorption ΔW over the propagating in waveguide energy W can be approximated as

$$\Delta W / W = \left(\text{Re}[\sigma] E_{\text{in}}^{2} \gamma 2\pi L_{\text{eff}} \Delta z\right) / \left(C \cdot S E_{\text{a}}^{2} V_{\text{gr}}\right) \tag{5}$$

The coefficient C is rather small ($C \sim \frac{1}{8\pi}$ for a cylindrical waveguide without absorption) and V_{gr} is of the order of the speed of light. For pristine graphene (5) can be simplified to

$$\Delta W / W \sim \gamma \frac{\Delta z}{\tilde{L}_{\mathrm{eff}}} g^2 \cdot \pi\alpha \,, \tag{6}$$

where $g = E_{\mathrm{in}} / E_{\mathrm{a}}$ represents the in-graphene-plane field weighted with respect to the average mode field and $\tilde{L}_{\mathrm{eff}} \approx \frac{S}{\pi L_{\mathrm{eff}}}$. It is easy to see that both $\tilde{L}_{\mathrm{eff}}$ and L_{eff} are of the order of $\lambda / |2n|$, where n is the mode effective index of the waveguide. This yields the relative light absorption per unit length induced by non-gated graphene as

$$A = \frac{10\log_{10}(1 - \Delta W / W)}{\Delta z} \sim \frac{10}{\ln(10)\tilde{L}_{\mathrm{eff}}} \gamma g^2 \cdot \pi\alpha \approx \frac{0.1\gamma g^2}{\tilde{L}_{\mathrm{eff}}} \,. \tag{7}$$

For perfect graphene coverage γ =1, the wavelength $\lambda = 1.5$ µm, n=1.5, and equal field contributions g =1, one should be able to achieve the modulation at the level of 0.2 dB/µm due to the presence of one graphene layer with the help of the effect of optical Pauli blocking. In reality, graphene covers only a part of a waveguide and the local in-plane fields in a typical plasmonic waveguide (i.e., with reasonable field confinement and propagation length) are smaller than the transverse, decreasing the above value by an order of magnitude and bringing it close to the inverse of typical propagation lengths in plasmonic waveguides. This alarming tendency compels one to analyse the possibilities offered by plasmonic waveguide configurations in more detail.

In the following, we consider several important plasmonic waveguide configurations, for which the modulation depth can be calculated more accurately.

A. Surface plasmon polariton (SPP) configuration (flat plasmons).

Additional absorption of energy ΔW on the length Δz per time Δt induced by a graphene flake can be evaluated as $\Delta W \approx \mathrm{Re}[\sigma] E_{\|}{}^2 \gamma \Delta y \Delta z \Delta t$, where Δy is a flake dimension perpendicular to the SPP propagation and $E_{\|}$ is the in-plane electric field, see (3). Neglecting electromagnetic energy in a metal film, we can write the mode energy flow as $W = \frac{n}{8\pi} c \Delta y \Delta t \int_0^{\infty} E_{\perp}^2(x) dx = \frac{nc\Delta y \Delta t}{8\pi} \frac{d_{\mathrm{p}}}{2} E_{\perp,0}^2$, where c is the speed of light, d_{p} is the field penetration depth in dielectric, $E_{\perp,0}$ is the perpendicular field at the surface of metal, and n is the refractive index of dielectric. This gives the following ratio of graphene absorption ΔW over the propagating in waveguide energy W: $\frac{\Delta W}{W} \approx \gamma \frac{4\pi\alpha}{n d_{\mathrm{p}}} \left(\frac{E_{\|}^2}{E_{\perp,0}^2} \right) \Delta z$ (graphene conductance was estimated as $\mathrm{Re}[\sigma] \approx \frac{e^2}{4\hbar}$). Using SPP properties, we write $d_{\mathrm{p}}^{-1} = \sqrt{k_{\mathrm{SPP}}^2 - n^2 k_0^2}$, $E_{\perp,0} = \frac{i k_{\mathrm{SPP}}}{\sqrt{k_{\mathrm{SPP}}^2 - n^2 k_0^2}} E_{\|}$ and obtain

$$\frac{\Delta W}{W} = \gamma \frac{4\pi\alpha}{n} \frac{\left(k_{\mathrm{SPP}}^2 - n^2 k_0^2\right)^{3/2}}{k_{\mathrm{SPP}}^2} \Delta z = \gamma \frac{4\pi\alpha}{n d_{\mathrm{p}}^3 k_{\mathrm{SPP}}^2} \Delta z \, . \tag{8}$$

The above equation can, in principle, be used for a quick estimation of the graphene absorption (and thereby maximum modulation depth by gating) in various plasmonic modulators.

For the case of poorly confined SPP modes this yields $\frac{\Delta W/\Delta z}{W} \cong \gamma \frac{8\pi^2\alpha}{\lambda}\left(\frac{\varepsilon_\mathrm{d}}{|\varepsilon_\mathrm{m}|}\right)^{3/2}$ and for the case of strongly confined modes (where contribution from the energy flow in the metal needs to be taken into account) we have $\frac{\Delta W/\Delta z}{W} \cong \gamma \frac{4\pi^2\alpha}{\lambda}\frac{N_\mathrm{SPP}}{n}$. For telecommunication wavelength λ=1.5 μm, $\varepsilon_\mathrm{d} \simeq 1$, $|\varepsilon_\mathrm{m}| \simeq 100$, full coverage $\gamma = 1$, and poorly confined SPP modes, we obtain $\frac{\Delta W/\Delta z}{W} \approx 4\cdot 10^{-4}$ μm^{-1}, which is even smaller than the SPP attenuation due to its absorption by metal (i.e., due to Ohmic loss) and gives the relative light absorption per unit length as $A = \frac{10\log_{10}(1-\Delta W/W)}{\Delta z} \approx \frac{10}{\ln 10}\frac{\Delta W/\Delta z}{W} = 1.7\cdot 10^{-3}$ dB/μm. This suggests that one can realistically achieve the values of modulation at telecom wavelengths produced by graphene optical Pauli blocking at the level of 0.002 dB/μm, but the perspectives for practical applications are rather bleak – the propagation length required for strong modulation is about the SPP propagation length (i.e., only a few percents of the launched SPP power can be efficiently modulated).

It is interesting to note that in the visible range, when the SPP modes are strongly confined so that $N_\mathrm{SPP} \approx 2n$ can be achieved, the relative absorption per unit length would reach $A \approx \frac{10}{\ln 10}\frac{\Delta W/\Delta z}{W} = \frac{10}{\ln 10}\gamma\frac{8\pi^2\alpha}{\lambda} \approx 4$ dB/μm. However, this absorption is again similar to the SPP absorption by metal and, in addition, it is not clear whether graphene can be gated to such degree that optical Pauli blocking can be extended to the visible light and hence whether this high absorption can be used for light modulation.

B. Cylindrical surface plasmon polariton configuration (CySPP) – full coverage.

For CySPP we may use the same arguments as for SPP and simplify calculations by adopting a view that CySPP is a planar SPP wrapped around a metal nanowire. This allows us to start with the same expression $\frac{\Delta W}{W} \approx \gamma \frac{4\pi\alpha}{nd_{\mathrm{p}}}\left(\frac{E_{\|}^2}{E_{\perp,0}^2}\right)\Delta z$ where for simplicity we assume the full coverage (γ=1). The penetration depth d_{p} can be evaluated as $d_{\mathrm{p}} \sim \varsigma\lambda$, where ς is a dimensionless coefficient which at telecom varies from 0.15 for a 20-nm radius nanowire to 0.5 for a 85-nm radius nanowire. The field component ratio can be again express via d_{p} and N_{SPP} as $E_{\perp,0} = \frac{ik_{\mathrm{SPP}}}{\sqrt{k_{\mathrm{SPP}}^2 - n^2k_0^2}} E_{\|} = id_{\mathrm{p}}k_0N_{\mathrm{SPP}}E_{\|}$ which yields

$$\frac{\Delta W / \Delta z}{W} \simeq \frac{\alpha}{n\lambda\pi\varsigma^3 N_{\mathrm{SPP}}^2}. \tag{9}$$

For telecom (λ=1.5 μm, $n \simeq N_{\mathrm{SPP}} \simeq 1$) this gives the relative absorption per unit length as $A \approx \frac{10}{\ln 10}\frac{\Delta W / \Delta z}{W} \simeq \frac{10}{\ln 10}\frac{\alpha}{\lambda\pi\varsigma^3} \approx 6.7\cdot 10^{-3}\varsigma^{-3}$ dB/μm. For reasonably large fully covered nanowires (of radius *r*~85 nm) $\varsigma = 0.5$, the modulation in a waveguide to be achieved by optical Pauli blocking of graphene is about 0.05 dB/μm. For much smaller nanowires (of radius *r*~20 nm) $\varsigma = 0.15$, the modulation depth could reach 2 dB/μm. Also in these cases, the achievable modulation depth is comparable with the CySPP absorption by metal.

C. Cylindrical surface plasmon polariton configuration (CySPP) – flat continuous graphene flake.

In the case of a flat graphene layer being placed on the top of a metal nanowire, the absorption of energy by graphene is given by a complicated integral:

$\Delta W = \mathrm{Re}[\sigma]\Delta z\Delta t\int_{-\infty}^{\infty} E_{\parallel}^{2}(y)dy$, where the in-plane field component is related (*contrary* to the above considered configurations) to the strong transverse CySPP field component projected on the graphene plane. Similarly to the above considered configurations, the mode energy flow can be estimated as $W = \frac{ncrd_{\mathrm{p}}\Delta t}{8}E_{\perp,0}^{2}$ (assuming that SPP is "wrapped" around the wire). Neglecting the contribution from the (relatively weak) longitudinal CySPP field component to the CySPP absorption by graphene, the above absorption integral can be evaluated as follows: $\Delta W = \mathrm{Re}[\sigma]r\Delta z\Delta t E_{\perp,0}^{2}\cdot\exp\left(-0.8\frac{r}{d_{\mathrm{p}}}\right)$. This gives $\frac{\Delta W/\Delta z}{W} = \frac{2\alpha}{nd_{\mathrm{p}}}\exp\left(-0.8\frac{r}{d_{\mathrm{p}}}\right)$, which results in the relative absorption per unit length as $A \approx \frac{10}{\ln 10}\frac{\Delta W/\Delta z}{W} \simeq \frac{10}{\ln 10}\frac{2\alpha}{n\varsigma\lambda}\exp\left(-0.8\frac{r}{d_{\mathrm{p}}}\right)$. For telecom wavelength, air cladding, *n*~1, and $R \ll d_{p}$, we obtain $A = 0.28 \div 0.08$ dB/μm, promising thereby to substantially exceed the CySPP absorption level.

It is necessary to stress that graphene gating requires a dielectric spacer between graphene and the metal which has thickness of *h*~50 nm in our experiments. The presence of a dielectric spacer moves graphene away from the enhanced electric fields of a plasmonic waveguide and could strongly reduce the achievable modulation depths. On the positive side, the supporting spacer layer can decrease the wrapping of a graphene layer around a nanowire or wedge.

To conclude this section, we note that the effect of metal surface curvature (wedge and nanowire SPPs with flat graphene flake) can provide plasmonic fields along the surface

of graphene and result in significant SPP modulation (using optical Pauli blocking) even at telecom wavelength and for SPP modes with a long propagation length.

2. AFM characterisation

To measure the thickness of hexagonal boron-nitride crystals (hBN) (which was used as a dielectric spacer for gating electrode) we conducted atomic force microscopy (AFM) measurements. Supplementary Figure 1**a** displays the topography of both hBN/Gr (Gr stands for graphene) and bare hBN on both the flat and nanostructured region for one of our plasmonic waveguides. An optical micrograph of the area, where the topography was measured, is shown in Supplementary Fig. 1**b**. Supplementary Figure 1**c** demonstrates the distribution of heights for the flat Au substrate, the flat Au substrate covered by hBN and the flat Au covered by hBN/Gr, black curve; as well as for the bare Au ridges, Au ridges/hBN and Au ridges/hBN/Gr, red curve. The peaks are affected by an aggregation of contaminants at the graphene-hBN interface, presumably hydrocarbons[4].

From these curves we can garner a step height of 66 ± 4 nm for hBN on the ridges. For the flat sections (covering Au, Au/hBN and Au/hBN/Gr, black curve) the individual peaks are wider than for the nanostructured region. This is probably due to an interface-dependent adhesion of hBN to the device whereby hBN (counterintuitively!) sits more flush on the ridges than it does on the flat gold. As a result, a larger step height of 72 ± 5 nm was measured for the section of hBN sitting on the flat gold. We used these measurements to evaluate the doping of graphene due to the applied backgate voltage. It is interesting to note that there is no periodicity apparent in the region of Au ridges/hBN(/Gr), which implies that hBN was thick enough to be rigid and flat.

3. DC resistivity of graphene flakes in the studied plasmonic waveguides

To check how graphene properties were affected by waveguide geometry, we performed routine measurements of DC resistivity as a function of gate voltage. Supplementary Figure 2 shows the resistivity data measured on one of our devices designed to allow for observation of charge carrier transport properties in separate regions: the planar section, Supplementary Fig. 2**a**, the nanostructured region, Supplementary Fig. 2**b**, and the device as whole, Supplementary Fig. 2**c**. The measurements were performed at room emperature in the dark. In each case there is some hysteresis (probably caused by migration of charges into dielectric[5]) and a peak at or close to $V_g = 0$ suggesting a shallowly-doped sample. The hysteresis is larger for the nanostructured region which could be expected due to high electric fields produced near the gold ridges. The zero voltage peak was generally observed at the condition of down-sweeping gating voltage (the graphene was grounded and the gating voltage was applied to the gold electrode below). It is worth noting that DC gating dependence of Supplementary Fig. 2**c** measured on the device as a whole (which includes both corrugated and flat regions) appears to be a weighted sum of gating dependence of resistivity for the flat region, Supplementary Fig. 2**a**, and that for the nanostructured region, Supplementary Fig. 2**b**. For the measurements performed along the whole width of the device, (Supplementary Fig. 2**c**), charge carrier concentrations of larger regions for both the nanostructured and flat sections were probed. Here the down-sweep peak and the up-sweep peak are almost coincident in V_g.

4. Raman Spectroscopy of the devices

A complimentary technique for assessing the level of doping comes in the form of gating-dependent Raman spectroscopy. In theory, the location of the charge neutrality point (CNP), which represents the level of doping, can be found from the gating dependent broadening of the G peak (or, alternatively, from the G-peak shift) induced by electron-phonon coupling[6]. Supplementary Figure 3**a** shows a part of typical Raman spectra of graphene comprising G-peak at three different gating voltages measured on one of our plasmonic waveguides. We have fitted the spectrum with a Lorentzian profile to find full-width-half-maximum of the peak and plotted it as a function of gating in Supplementary Fig. 3**b**. The change of FWHM produced by gating can be recalculated into the shift in Fermi energy[7] shown in the top axis of Supplementary Fig. 3**b**. This allows us to estimate the gating capacitance as ~ 60 nF/cm^2 which suggest that the thickness of hBN is about ~ 60 nm and compares well with the AFM measurements (h = 52nm for this sample; data not shown). Hence we need to apply voltage at the level of ~10-16V (for various devices) in order to shift Fermi energy from $E_F = 0$ to $E_F = 0.4$ eV and achieve complete optical Pauli blocking for telecom wavelengths.

5. Wedge plasmon polaritons

Wedge plasmon polaritons (sometimes also called wedge plasmons (WP) for brevity) are the eigenmodes of metal wedges, where mode localization arises from the increased interaction of the surface plasma oscillations on the adjacent sidewalls. WP have been studied, both theoretically and experimentally, for triangular wedges[8-10], but they can exist at any wedge geometry, in particular at the edge of a planar gold structure. Due to the mode symmetry, it cannot be excited with linearly polarized light with polarization perpendicular to the wedge axis. However, using wedge roughness or (preferably) a grating and light polarized

along the wedge axis, one can match the longitudinal electric-field component of the mode to achieve its excitation. In our experiments, we used the same grating that was fabricated for excitation of flat and corrugated plasmons, since the ridges of the grating were made up to the very edge of the planar gold structure. The period of the grating did not match exactly the mode effective index (it was optimized for excitation of flat plasmons), but it was nevertheless easy to excite the mode using tightly focused laser beam (spot size ~ 5 μm).

If the planar gold structure is fabricated on a transparent substrate with refractive index value above the effective index of the mode, observation of confined WP mode becomes possible in the far field using leakage-radiation microscopy[11] (LRM) due to leakage of the mode into the substrate (Supplementary Fig. 4**a**). Finite-difference time-domain modelling supports this conclusion (Supplementary Fig. 4**b**). We used LRM to make preliminary characterization of plasmonic structures before fabricating graphene modulators. This technique allows easy observation of confined plasmonic modes (Supplementary Fig. 4**d**). Moreover, with the access to Fourier plane of the image, one can make spatial-frequency filtering[11] to suppress directly transmitted and scattered light (Supplementary Fig. 4**e**). Analysis of the Fourier-plane image of LRM is a powerful tool for mode characterization. Thus, a straight line in the Fourier image (inset on Supplementary Fig. 4**d**) is a typical feature of waveguided (confined) modes[12], and hence indicates that the mode observed is indeed the propagating WP mode rather than just scattering of the laser beam by the metal edge. The two lines correspond to two WPs propagating in opposite directions away from the excitation point, and different brightness of the lines in the upper and lower parts of the Fourier image are due to the asymmetric nature of the metal edge. The dim circle of the largest diameter corresponds to the angle of total internal reflection (TIR), since it is the direction of maximum intensity radiated by a dipole near a planar interface[13]. From this, one can evaluate the effective mode index by measuring the distance of the straight lines from the image center

normalized to the radius of the TIR circle. We estimated the WP mode effective index to be 1.10 ± 0.05.

Note that LRM was used only for preliminary characterization of the structures, whereas measurements of the modulation depth were accomplished by collecting the light scattered on the out-coupling slits. Since excitation of WP mode also leads to simultaneous excitation of FP mode (which is evidenced by the glow of the slits in the vicinity of the edge, Supplementary Fig. 4**d**), the values of WP modulation are underestimated. Indeed, the detected modulated WP signal is mixed with FP, which is modulated by a substantially lower degree.

We also would like to point out that plasmonic waveguides with sharp features can provide an extremely strong local field enhancement, which is not necessarily accompanied by strong confinement of the total electromagnetic energy[1]. In our case, the confinement of the WP depends primarily on the gold-film thickness. This geometry is therefore of great advantage for graphene-based modulator, because it provides a good overlap of graphene with in-plane electric field of the WP mode. The exact overlap is difficult to predict, since it depends on the profile of the metal edge and the shape of the BN and graphene layers wrapping the wedge. However, a qualitative image can be obtained from finite-element modelling of the structure with realistic BN layer curvature parameters (Supplementary Fig. 4**f**). Note that the length of the red arrows on the figure is proportional to the local electric-field strength, and the direction of arrows coincides well with the graphene plane in the substantial part of the mode volume.

6. Operation of hybrid graphene plasmonic waveguide modulators

Here we present results taken from two different non-encapsulating devices. Supplementary Figure 5 shows data for the flat-plasmon (FP) and (CP) modes: open symbols correspond to a sample with thickness $t \approx 70$ nm, nanostructure ridge width of $w = 200$ nm and device length $l = 27$ nm; filled symbols correspond to a sample with $t \approx 50$ nm, $w = 300$ nm and $l = 75$ nm. Both structures have nanostructure ridge width of 600 nm.

We found that a non-zero modulation depths for CP-mode on one device were obtained only for negative V_g^{dc}, see light blue filled circles of Supplementary Fig. 5**a**. This behaviour is most probably related to photo-induced doping in graphene[14] and suggest that strong ambient light can affect the operation of graphene modulators. Another undesirable property of non-encapsulating devices is the fact that they could sometimes show strong hysteresis in optical response. For example, reproducible data with symmetrically increasing FP-mode modulation depth about a V_{cnp} was observed for one device (filled green triangles). At the same time, for the other device, a measureable modulation depth would be unobservable one day (light-brown triangles, open) and yet observed the next day (light-brown triangle, crossed), Supplementary Fig. 5**b**. We attribute this behaviour to sensitivity of the devices to local conditions which can be solved by device encapsulation. Generally, device operation was also sensitive to processing conditions. For the FP channel of the modulator, in particular, any roughness in the modulator morphology introduced during processing would impact in the orientation of graphene with respect to the electric field, which would change the coupling of graphene to the field and explain such a difference in optical response between devices.

Supplementary References

1 Oulton, R., Bartal, G., Pile, D. & Zhang, X. Confinement and propagation characteristics of subwavelength plasmonic modes. *New J Phys* **10**, 105018 (2008).

2 Maier, S. A. *Plasmonics: Fundamentals and Applications*. (Springer, 2007).

3 Maier, S. A. Plasmonic field enhancement and SERS in the effective mode volume picture. *Optics Express* **14**, 1957-1964, doi:10.1364/oe.14.001957 (2006).

4 Haigh, S. *et al.* Cross-sectional imaging of individual layers and buried interfaces of graphene-based heterostructures and superlattices. *Nat Mater* **11**, 764-767 (2012).

5 Wang, H., Wu, Y., Cong, C., Shang, J. & Yu, T. Hysteresis of electronic transport in graphene transistors. *ACS Nano* **4**, 7221-7228 (2010).

6 Pisana, S. *et al.* Breakdown of the adiabatic Born-Oppenheimer approximation in graphene. *Nat Mater* **6**, 198-201, doi:10.1038/nmat1846 (2007).

7 Das, A. *et al.* Monitoring dopants by Raman scattering in an electrochemically top-gated graphene transistor. *Nature Nanotechnology* **3**, 210-215, doi:10.1038/nnano.2008.67 (2008).

8 Pile, D. F. *et al.* Theoretical and experimental investigation of strongly localized plasmons on triangular metal wedges for subwavelength waveguiding. *Applied Physics Letters* **87**, 061106 (2005).

9 Moreno, E., Rodrigo, S. G., Bozhevolnyi, S. I., Martín-Moreno, L. & García-Vidal, F. Guiding and focusing of electromagnetic fields with wedge plasmon polaritons. *Physical Review Letters* **100**, 023901 (2008).

10 Boltasseva, A. *et al.* Triangular metal wedges for subwavelength plasmon-polariton guiding at telecom wavelengths. *Optics Express* **16**, 5252-5260 (2008).

11 Drezet, A. *et al.* Leakage radiation microscopy of surface plasmon polaritons. *Materials science and engineering: B* **149**, 220-229 (2008).

12 Radko, I. P., Fiutowski, J., Tavares, L., Rubahn, H.-G. & Bozhevolnyi, S. I. Organic nanofiber-loaded surface plasmon-polariton waveguides. *Optics Express* **19**, 15155-15161 (2011).

13 Novotny, L. & Hecht, B. *Principles of nano-optics*. (Cambridge university press, 2012).

14 Ju, L. *et al.* Photoinduced doping in heterostructures of graphene and boron nitride. *Nature nanotechnology* **9**, 348-352 (2014).
